# Characterisation of sensitivity and orientation tuning for visually responsive ensembles in the zebrafish tectum

**DOI:** 10.1038/srep34887

**Published:** 2016-10-07

**Authors:** A. W. Thompson, E. K. Scott

**Affiliations:** 1 School of Biomedical Sciences The University of Queensland, St Lucia, QLD, 4072, Australia; 2 The Queensland Brain Institute, The University of Queensland, St Lucia, QLD, 4072, Australia

## Abstract

Sensory coding relies on ensembles of co-active neurons, but these ensembles change from trial to trial of the same stimulus. This is due in part to wide variability in the responsiveness of neurons within these ensembles, with some neurons responding regularly to a stimulus while others respond inconsistently. The specific functional properties that cause neurons to respond more or less consistently have not been thoroughly explored. Here, we have examined neuronal ensembles in the zebrafish tectum responsive to repeated presentations of a visual stimulus, and have explored how these populations change when the orientation or brightness of the stimulus is altered. We found a continuum of response probabilities across the neurons in the visual ensembles, with the most responsive neurons focused toward the spatial centre of the ensemble. As the visual stimulus was made dimmer, these neurons remained active, suggesting higher overall responsiveness. However, these cells appeared to represent the most consistent end of a continuum, rather than a functionally distinct “core” of highly responsive neurons. Reliably responsive cells were broadly tuned to a range of stimulus orientations suggesting that, at least for this stimulus property, tight stimulus tuning was not responsible for their consistent responses.

The natural world presents a practically limitless diversity of sensory stimuli, and perceiving and interpreting these stimuli is one of the nervous system’s most important and challenging tasks. Given the generally high intrinsic noise and broad tuning preferences of individual neurons, the information that can be encoded by single neurons is insufficient for the fine discrimination of stimuli that animals achieve^1^,^2^,^3^,^4^,^5^. The idea that cells form assemblies to encode information as a population was proposed more than 50 years ago^6^, suggesting that integration of synchronous activity from populations of neurons underlies the enormous breadth observed in encoding capacity^7^,^8^,^9^,^10^. In support of this theory, synchronous activity has been found in localised populations of cells in the brains of various organisms in response to visual^11^,^12^,^13^, auditory^14^,^15^,^16^,^17^, somatosensory^18^,^19^,^20^,^21^ and olfactory stimuli^22^,^23^,^24^,^25^, as well as during exploratory behaviours^26^,^27^. This implies that concurrent activation of populations of neurons within the brain is a fundamental method by which information is encoded (see also selected reviews^28^,^29^,^30^,^31^,^32^,^33^). However, while several recent studies have reported on ensembles of cells responsive to visual stimuli, variability has been observed in the cellular composition of these ensembles across trials presenting the same stimulus^34^,^35^,^36^,^37^,^38^. This variability means that many of the cells present in a stimulus-specific ensemble are active in some trials and inactive in others, and this could undermine the ensemble’s ability to report reliably on the stimulus that it encodes. The factors that lead to this variability have not been thoroughly explored, and its functional significance remains unexplained.

If ensembles contribute to sensory processing, then understanding the response properties of their constituent neurons is important. Our past work, and that of others, has suggested that consistently responsive neurons, termed “core cells”, may represent a small population of cells whose properties set them apart from the remainder of the ensemble^36^,^38^,^39^. Because they are consistently responsive across trials, they may signify a focal population of cells selective to specific stimuli. If this were true, identifying their relationship with the rest of the ensemble, and establishing the roles of non-core cells in encoding stimuli would be important next steps. It is possible that they simply represent the neurons that are best tuned to a specific stimulus. Alternatively, they may be cells that act to encode broad stimulus features, while less reliable cells may have other roles such as reporting on the stimulus context or current network state^40^, or discriminating fine stimulus features^41^. Finally, their consistency may simply reflect higher overall responsiveness, thus subserving the detection of less salient stimuli.

The heterogeneity of response reliability between different cells in an ensemble is of considerable interest and is deeply important for understanding how information can be reliably encoded in the brain. With the goal of defining these properties of neural ensembles, we have observed large populations of individually-resolvable neurons in the zebrafish tectum through numerous trials of a consistent visual stimulus. First and foremost, this was intended to reveal the range of response probabilities present among the neurons in the observed ensembles. Specifically, we aimed to determine whether a “core” population of neurons exists that can be defined by their consistently reliable responses, and are functionally separable from the rest of the ensemble based on their response properties. Regardless of whether core cells are a distinct category, the question remains as to what functional characteristics cause some cells to respond more consistently than others. To address this, we have looked at the changes in ensemble composition over a range of stimulus intensities and orientations. These experiments aim to reveal the relative contributions made by baseline responsiveness, stimulus specificity, and tuning breadth, to the probability of each neuron’s firing in a given trial, and therefore to the functional composition of visually-responsive ensembles in the zebrafish tectum.

## Results

**Figure 1:**
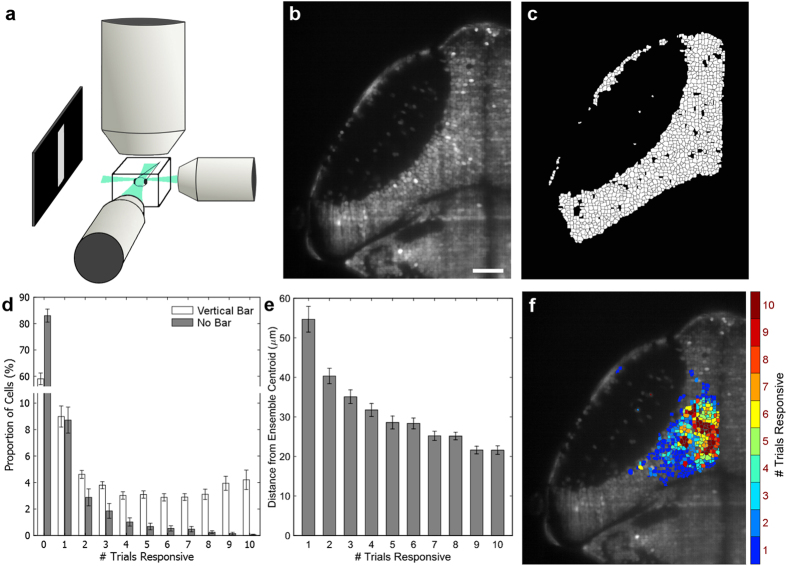
Identification of the most consistently responsive cells to a visual stimulus. **(a)** 6 dpf *Elavl3:H2B-GCaMP6f* larvae were presented with bright vertical bars moving caudal-to-rostral, and their contralateral tecta were imaged using SPIM with two orthogonal illumination planes. **(b)** Example image of nuclear GCaMP6f expression in the left tectum. Scale bar = 35 μm. **(c)** Automated segmentation of individual ROIs from the image in panel b. **(d)** When presented with a visual stimulus ten times, the number of trials during which cells were active increased relative to animals presented with no stimulus (spontaneous activity) (p = 8.00 × 10^−5^ versus slope = 0, linear regression, n = 18 fish, 801 ± 19.6 cells per fish, error bars = SEM). **(e)** Cells that were more consistently responsive were, on average, located closer to the ensemble centroid. **(f)** The spatial distribution of cells with different response consistencies to the visual stimulus, from a single representative larva. Highly reliable cells are located near the centre of the cluster, and the consistency of these responses gradually declines toward the edges.

We have observed population responses in the zebrafish larval tectum to a baseline stimulus, a moving bar, and described how the responsive ensembles change as this stimulus is gradually varied in brightness and orientation. We approached this questions using larvae with pan-neuronal expression of nuclear-localized GCaMP6f^42^ and selective plane illumination microscopy (SPIM, Fig. 1a–c). The reliability with which cells responded to ten presentations of the stimulus varied greatly across neurons in the tectum (Fig. 1d). During control trials without a visual stimulus, spontaneous activity was present at a low, consistent rate, leading to a small population of neurons that responded one or a few times. However, visually responsive neurons showed a wide range of consistency, from neurons responding once or twice to those responsive to all ten presentations of the stimulus (Fig. 1d). Given the spatially organized receptive fields of tectal neurons within the retinotectal map^34^,^43^,^44^, a spatially consistent ensemble would be expected for a stimulus repeatedly presented at one location within the visual field. Beyond this, the level of the neurons’ consistency was reflected in the spatial structure of the responsive ensemble, in that the most consistently responsive cells were located closest to the centroid of the ensemble (p = 3.36 × 10^−4^ versus slope = 0, linear regression) (Fig. 1e).

These centrally located, regularly responsive neurons (Fig. 1f) are the cells composing what has previously been reported as the ensemble’s core. Since cells towards the periphery of the responsive ensembles may have receptive fields that only partially intersect with the visual stimulus, the inconsistency of responses seen in these cells may be expected. However, cells nearer to the spatial centres of these ensembles still exhibit a range of response reliability. Viewing the ensemble as a whole, we observe a continuum across the entire range of response probabilities (Fig. 1d). These observations also held true when we increased our minimum response threshold for inclusion in the dataset from 10% ΔF/F to 20% ΔF/F (Supplementary Fig. 1). As such, we regard the nine- and ten-time responders as simply the most reliably responsive neurons, rather than a functionally distinguishable core. Nonetheless, the roles of these consistently responsive neurons within the ensemble, and the characteristics that drive their reliability, are of interest to the functional architecture of the ensembles.

**Figure 2:**
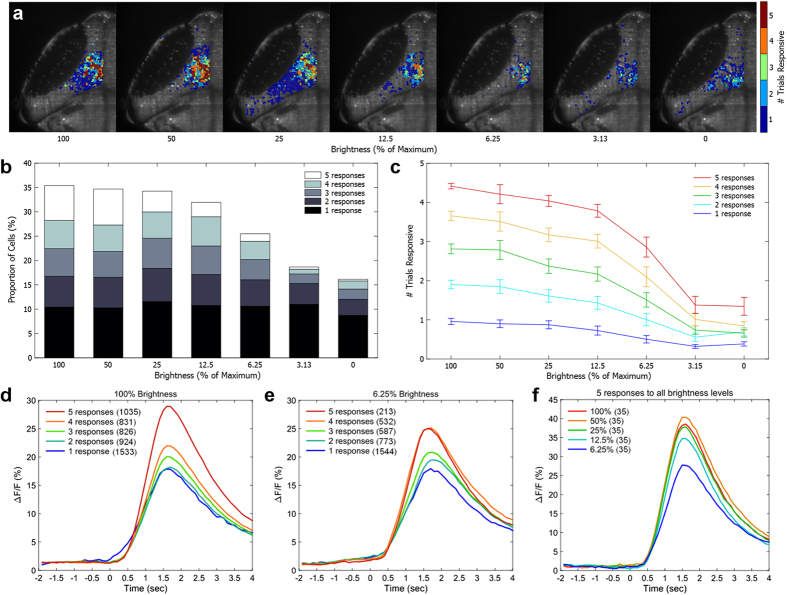
Consistently-responsive cells remain more responsive than other cells as stimulus intensity is reduced. **(a)** Example distribution of tectal cells responding to a moving vertical bar of decreasing brightness levels, drawn from a single representative larva. Cells more reliably responsive to bright bars were also more likely to be responsive to faint bars. **(b)** The proportion of total cells active in any trial, including the proportion of consistently-responding cells, decreased as stimulus brightness was decreased to 0 (no stimulus). **(c)** Cells responsive at different initial rates (taken from a separate set of five trials at 100% brightness, legend in **c**) decreased the number of trials to which they were responsive as stimulus brightness was decreased. **(d)** Reliably responding cells at 100% brightness had stronger ΔF/F responses than those responding less consistently to the same stimulus. **(e)** This pattern was maintained at 6.25% brightness, albeit with fewer cells, and responses were of a similar strength to those in the 100% brightness condition. **(f)** Cells that responded to all trials at all intensities from 100% to 6.25% showed a decrease in response strength when responding to fainter stimuli. In d–f, the number of cells in each category is indicated in parentheses.

To test the sensitivity of neurons with different response probabilities, we decreased the brightness of the vertical bar stimulus while maintaining a black background, thereby decreasing its contrast (Fig. 2a,b). Out of a desire to avoid habituation through numerous trials, and because nine- and ten-time responsive neurons mapped similarly in our initial analysis, we reduced these experiments to five trials per stimulus (although we presented 10 trials at 100% brightness, five to establish the response properties of all neurons, and five more as part of the brightness gradient). We observed a similar number of responsive neurons at 100%, 50%, and 25% of original stimulus brightness, but this decreased at 12.5% brightness, and decreased significantly at 6.25% brightness (27.95% decrease from 100% brightness, p = 0.001, paired t-test) (Fig. 2a,b). This was accompanied by a significant reduction in the proportion of cells consistently responding to dimmer stimuli. For example, the number of cells responding five out of five times decreased by 58.87% and 78.37% at 12.5% and 6.25% brightness, respectively (p < 0.01, paired t-test) (Fig. 2b, see also Supplementary Fig. 2a). Notably high levels of spontaneous activity within the ensemble also led to responses to the stimuli at 3.125% and 0% brightness (no stimulus). This may be a result of entrainment in these cells following the high number of repeated stimulus presentations as previously described in the zebrafish tectum^37^. However, the fact that the cells most reliably responsive to the 100% brightness condition have a higher level of spontaneous activity than less reliable cells during trials without a stimulus (p < 0.05, paired *t*-test, Fig. 2c) suggests that these cells may have lower physiological thresholds to fire, or other properties that cause them to be generally more active.

If reliably responsive cells have low perceptual or physiological activation thresholds, they may be relatively enriched in ensembles responding to dimmer stimuli. Cells that responded at different rates during the original (100% brightness) condition all decayed in their likelihood of firing as the stimulus brightness was decreased (Fig. 2c). However, while cells that responded five out of five times at 100% brightness were no longer as consistent at 6.25% brightness, 92.7% (±3.7%) of them responded in at least one trial. By comparison, only 33.9% (±4.9%) of single-trial responders showed a response to any of the 6.25% brightness trials (Supplementary Fig. 3). The remaining cells, responsive two, three, or four times at 100% brightness, showed a smooth decline in responsiveness to dimmer stimuli, graded to their original responsiveness.

To address the possibility that stimulus intensity is encoded in neurons’ response profiles, in addition to the number of responsive cells, we explored the relationships among stimulus strength, response probability, and the profiles of observed responses. We found that neurons with a higher likelihood of responding to the 100% stimulus tended towards stronger peak responses, as measured by their change in GCaMP6 fluorescence (Fig. 2d). The same effect was observed for the 6.25% stimulus, and the response strength was similar between 6.25% and 100% stimuli (Fig. 2e). Finally, we examined responses from the very most responsive neurons, those that responded five times to each of the stimuli from 100% to 6.25% (25/25 responses, total). These cells had similar response intensities compared to other consistently-responsive neurons in the 100% or 6.25% conditions, but still showed graded responses to different stimulus intensities relative to the original brightness condition (−9.68% and −26.65% peak ΔF/F at 12.5% and 6.25% brightness respectively; p = 0.006 and 4.1 × 10^−14^ respectively, paired t-test) (Fig. 2f).

These results suggest that neurons that are most consistently responsive to bright stimuli also have relatively low thresholds for responding to dim stimuli, and encode aspects of stimulus strength or contrast in their responses. The data also reveal a continuum of response profiles within the ensemble’s neuronal population, rather than a sharp delineation between core and non-core cells. As such, while ensembles will contain cores for particular stimuli based on a given response criterion set by the experimenter (as we have described previously^38^), these cores do not represent a functionally distinct population within the ensemble. Their higher calcium signals with strong stimuli indicate that low-threshold cells have higher activity levels than their higher-threshold, less consistent counterparts even when the latter population reaches a perceptual threshold. In other words, consistently responsive neurons show lower thresholds both for generating a minimal response, and for producing more robust activity.

**Figure 3:**
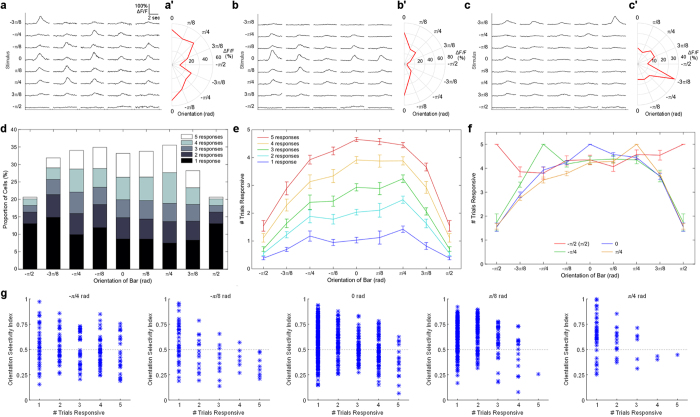
Consistently responsive cells do not encode stimulus orientation. **(a)** Example calcium response traces for a broadly tuned cell across all trials and all orientations tested. **(a′)** Average response amplitudes for the cell in panel (**a)** show relatively broad tuning for all orientations tested (except the π/2 condition). **(b,b′)** Responses as per panels (**a**,**a′)** for a cell specifically tuned to a moving bar with vertical orientation (orthogonal to direction of motion). **(c,c′)** Responses for a cell specifically tuned to a moving bar with a non-vertical orientation (rotated −3π/8 rad relative to vertical). **(d)** The proportion of total cells active in any trial, and rate of consistently responsive cells, remained relatively consistent to most stimuli as the orientation of the moving bar was rotated around π radians (180 degrees). However, when the bar was horizontal (-π/2 or π/2 condition), no caudal-to-rostral motion existed in the stimulus, and the number of responsive cells was reduced. **(e)** Cells responsive to the vertical (0 rad) bar at different rates showed a very gradual decline in response consistency at orientations away from 0, suggesting that cells responding at all levels of reliability are broadly tuned to orientation. **(f)** Cells responding to all trials for stimuli at orientations other than vertical were broadly tuned to stimulus orientation, with a slight bias towards the 0 rad condition (orthogonal to the direction of movement). **(g)** The OSI for cells most selective at each of five different stimulus orientations between -π/4 and π/4, plotted against the in which the cells are number of trials at each of these orientations responsive. Cells with an OSI above 0.5 (grey dotted line) are considered to be selective for a given orientation. Reliably responsive cells are generally less likely to be selective for a particular orientation.

The consistency of some cells’ responses may be explained by lower activation thresholds, but it is possible that they are also particularly well tuned to the stimulus that we have presented: a moving vertical bar. In order to test this, we changed the orientation of the bar, while maintaining its caudal-to-rostral movement across the visual field. Over the five trials with each orientation tested, we observed cells with a range of response profiles. Most were broadly tuned to different orientations (Fig. 3a,a′), while others showed preferential tuning to the vertical bar (Fig. 3b,b′) or specific orientations away from vertical (Fig. 3c,c′). If a large proportion of the consistently responsive cells were also orientation specific, and if tuning to these specific orientations were driving their responses, we would expect abrupt changes in membership of the consistent population within our ensembles as the orientation shifts away from vertical.

Across our population of responsive neurons, as stimulus orientation was shifted in either direction, there were no dramatic changes in the overall numbers of active cells, or their response rates compared to the original, 0 radian (rad) stimulus (Fig. 3d, see also Supplementary Fig. 2b). The only exception to this was in the π/2 rad condition (horizontal bar), in which the larva would perceive no motion as the bar moves from caudal to rostral. This supports previous findings that tectal cells, particularly the reliably responsive cells described here, are more sensitive to stimuli in motion. When we examined cells that responded to all trials in the vertical bar (0 rad) condition, we found only a shallow decline in the response rate of these cells to different stimulus orientations (Fig. 3e, red line). These cells were still responding, on average, more than four out of five times to stimuli offset π/4 rad from the original stimulus, with no significant difference in response rates for stimulus orientations between - π/4 and π/4 (p > 0.05, ANOVA with Tukey-Kramer correction). Similarly broad tuning was seen for less consistent cells responding to the original stimulus (Fig. 3e), as well as for consistent cells responding to bar orientations other than vertical (Fig. 3f). Here, the consistency of cells that responded to all five trials at a particular orientation was mapped for all other orientations. The response profiles of these cells were nearly indistinguishable from one another, except for the (inevitable) high values at the orientation for which each consistently-responsive population was calculated (Fig. 3f). This suggests that, at least for tectal neurons responding to bright moving bars on a dark background, consistently responsive cells are broadly tuned to bar orientation, and that the consistency of their responses is not based on a precise tuning to a specific stimulus orientation. To confirm this, we examined the orientation selectivity index (OSI) for all responsive cells and determined the stimulus orientation to which they are most selectively responsive. We found that for each preferred stimulus orientation, reliably responsive cells were less likely to be orientation selective (>0.5 OSI) (Fig. 3g). This reinforced our conclusion that response consistency of the reliably responsive population of cells is not due to the fact that they are specifically tuned to the stimulus orientation presented.

To explore this interpretation further, we looked at the identities of consistently responsive cells presented with differently oriented bars. We calculated the matching index (MI), described previously^44^, to determine the ratio of cells within one ensemble that were also present in a second ensemble. We observed a smooth decline in the MI between the consistently responsive cells in the 0 rad condition, and those in ensembles responding to orientations diverging from 0 (Supplementary Fig. 4, top row). Since we did not observe any abrupt changes in the composition of these groups, this further supports the conclusion that consistently responsive cells do not form a unique functional unit specifically responding to a particular orientation. Finally, we aimed to determine whether the low-threshold cells that remained responsive as the stimulus brightness was decreased were the same population of cells that remained responsive as the stimulus orientation was rotated. We did this again by calculating the MI comparisons between reliably responsive cells for ensembles responsive to each of these different conditions. While we did not present all 48 stimulus combinations, we were able mathematically to compare the populations of cells responsive to these different pairs of stimuli. We found the MI declined with comparisons between ensembles of both altered brightness and orientation (Supplementary Fig. 4). However, this decline fit with a linear combination of the decline in the MI for deviations in only brightness or orientation away from the original 100% brightness, 0 rad stimulus (R^2^ > 0.5). Based on the MIs of these comparisons, we determined that these low threshold cells were indeed likely to represent the same population of consistently responsive cells as stimulus orientation was rotated.

## Discussion

It is well established that ensembles of co-active neurons are central to sensory processing, among other neural computations. This has been explored for smaller numbers of neurons through repeated presentations of sensory stimuli paired with electrophysiology^11^,^12^,^13^,^14^,^15^,^16^,^17^,^18^,^19^,^20^,^21^,^23^,^24^,^25^,^26^,^27^,^35^, and also at the population level, principally through calcium imaging^22^,^34^,^36^,^37^,^38^,^39^,^40^,^44^. With the rise in genetically-encoded calcium indicators, a number of groups have taken advantage of the zebrafish model system to show how synchronous activity in the tectum^34^,^38^,^44^,^45^, habenula^46^,^47^, midbrain^48^ and spinal cord^49^,^50^,^51^ is spatially organised, and how patterns of synchronous activity relate to sensory processing. A thorough understanding of the functional architecture of responsive ensembles, however, requires that activity in an entire population of neurons be tracked through numerous presentations of a single stimulus. This is necessary to identify the response probabilities of neurons throughout the ensembles. Once individual neurons’ responses to a specific stimulus have been determined, altered stimuli can be used to explore the causes of these response characteristics.

Using this approach, we have shown that there is a spectrum of sensitivities for neurons in the larval zebrafish tectum, resulting in a range of response probabilities to a visual stimulus. Our data from experiments with up to ten stimulus presentations show no evidence of a bimodal distribution of response probabilities. This means that we have observed neurons with a continuum of response probabilities, suggesting that the ensembles do not contain distinct and separable “cores” of highly responsive neurons. This is in contrast to our prior conclusions^38^, and those of others^39^, which were drawn from experiments with fewer repetitions of the stimulus, and therefore a reduced ability to map out the continuum of responses present in the ensembles.

The activity in the responsive ensembles appears to encode stimulus intensity primarily through the number of active cells, with highly-responsive cells remaining active (although less consistent) at low stimulus intensities. Beyond this, there may be an element of rate coding, especially among the more responsive neurons, with stronger stimuli being represented by larger calcium transients. The circuits formed among tectal neurons are not well understood, but these results point toward a system in which the overall activity in a regionalized ensemble of neurons (a function of the number of responsive cells, and to a lesser degree, the strength of their responses) represents the strength of the perceived stimulus.

Rate coding is often cited as the primary means of encoding stimulus strength in mammals for various senses including visual^52^,^53^,^54^, auditory^55^,^56^ and tactile^57^,^58^,^59^, but the size and composition of ensembles can also play an important role in these higher vertebrates. For example, whisker deflections are represented in the barrel cortex of rodents as an ensemble response^57^,^58^. Similar to the results presented here, the proportion of cells responsive in the ensemble relies significantly on stimulus strength^57^, while the shape and spatial profile of these ensembles remains unchanged^58^. This suggests that the ensemble properties described here, and the factors related to response consistency, may be shared with other neural systems across phylogeny.

Stimulus features unrelated to intensity, such as the different orientations tested in these experiments, are also likely to influence the ensembles’ responses. However, we found that virtually all cells, regardless of their baseline response probability (Fig. 3e) or optimal stimulus angle (Fig. 3f), had a similar chance of responding to most different bar orientations. However, we also found that cells with higher response consistency at a given orientation were less orientation selective (Fig. 3g). This broad tuning argues against the idea that consistently responsive neurons have high specificity for the stimulus being presented, or that these cells form discrete functional units selectively encoding unique stimulus features. However, these cells could be highly tuned to stimulus features not examined in the present study, such as the speed^45^,^60^,^61^,^62^ or direction^43^,^44^,^45^,^61^,^63^ of motion, or the size^43^,^44^,^45^,^60^,^62^,^64^,^65^ or shape^43^ of the stimulus. Indeed, the significant drop in response reliability to visually-stationary horizontal bars suggests that these cells are highly selective for stimuli in motion. Therefore, while the orientation of the bar seems to have little influence on the structure of the ensemble, we cannot rule out contributions from other stimulus features.

Interestingly, just as the identities of consistently responsive neurons diverge slowly in the zebrafish tectum as stimulus orientation is rotated (Fig. S3), so too do those of the cells responsible for encoding information in more complex systems. For example, Leutgeb and colleagues^66^ have investigated ensemble responses from the hippocampus of rats. When the shape of the rat’s enclosure is gradually morphed between two learned configurations, they observed a gradual shift in the activity of CA3 networks responsible for spatial recognition. This suggests that a common feature of sensory processing may be the abilty to encode and identify subtle differences in information within the distributed activity of populations of cells without shifting between unique functional circuits, which may be important for the perception of spatially- and temporally-continuous stimuli.

These results raise the obvious question of how, if the ensemble responses are similar, do these larvae distinguish bars of different orientations. One possibility is that they do not. The stimuli presented here may not be sufficiently different for the larvae to distinguish them. This seems unlikely, given the strong evidence for visual orientation sensitivity across numerous studies in an array of model systems^12^,^67^,^68^,^69^,^70^. Another explanation is that a small number of neurons tightly tuned to orientation (such as those seen in Fig. 3b,c) may encode this information, but that their signals are lost among the large number of broadly tuned neurons in our population-level analysis. Terminals from orientation-selective retinal cells are distributed across the rostro-caudal extent of the tectum with some slight preference for either pole^69^,^71^, and the recipient orientation-selective tectal cells also appear to be scattered broadly across the periventricular zone^68^. Since we stimulated only a small portion of the central visual field in these experiments, and orientation selective cells respond less consistently, we would only expect to elicit responses in a relatively small number of these cells in any given trial. Indeed, as Hunter and colleagues^68^ only observed an average of 5–6 orientation-selective periventricular neurons per animal while stimulating more than 30 degrees of the visual field, we would expect such cells to account for a small minority of the neurons in the ensembles that we describe here. Deeper analyses across a range of orientations will be necessary in the future to discern among these possibilities, and to reveal exactly how given ensembles represent the specific stimuli to which they respond.

In summary, the results presented here demonstrate that, in the zebrafish larval tectum, ensemble structure is based primarily around the overall responsiveness of each of its constituent neurons. Tectal neurons exhibit a wide range of responsiveness levels, which evidently occur in a continuum, including neurons that respond consistently to a wide range of stimuli. Tight stimulus tuning at least for the differently oriented bars presented in this study, is not a major factor in the composition of these ensembles, suggesting that a more subtle form of coding is responsible for distinguishing closely related stimuli.

## Methods

### Animals

All procedures were performed with approval from The University of Queensland Animal Welfare Unit (in accordance with approval SBMS/305/13/ARC). Zebrafish (*Danio rerio*) larvae were maintained at 28.5 °C on a 14 hr ON/10 hr OFF light cycle. Adult fish were maintained, fed, and mated as previously described^72^. All experiments were carried out in *elavl3*:*H2B-GCaMP6f* larvae^42^,^73^, kindly provided before publication by Misha Ahrens at the Howard Hughes Medical Institute (HHMI), Janelia Farm Research Campus (Ashburn, VA, USA).

### Imaging tectal activity

6-day post-fertilization (dpf) larvae of the transgenic strain *elavl3:H2B-GCaMP6f* were immobilised dorsal side up in 1.5% low melting point agarose (Progen Biosciences, Australia). Larvae were then transferred to custom-made, glass-walled imaging chambers and allowed to acclimate for 20 minutes prior to imaging under 488 nm illumination on a custom-built selective plane illumination microscope (Table S1). Imaging larval zebrafish with 488 nm light using a selective-plane illumination method has been previously shown to reduce or abolish some visual responses^74^, therefore we tuned the intensity of our plane to a level that still permitted robust visual responses. While we cannot rule out a reduction in the sensitivity of our assay, any artefacts arising from direct stimulation of the eye by the plane should be uniform across the dataset, such that they would not be expected to produce spurious results.

A single tectal hemisphere was imaged at 10 Hz for five consecutive trials at 75 μm below the first visible tectal cell body. This plane was chosen as it was responsive to most visual stimuli in previous experiments^38^. In each trial, larvae were presented with sixteen visual stimuli, delivered at 20 second intervals. The order of stimulus presentation was randomised between fish, but not between experimental trials. Trials were separated by 90 seconds of a blank screen with no visual stimuli.

The sixteen visual stimuli were presented contralateral to the tectum being imaged on a 7 × 5 cm LCD screen positioned 8 cm from the larva, covering approximately 50 × 35° of the visual field. The primary stimulus presented was a bright, 4° wide vertical bar on a dark background, moving from rostral to caudal across 25° of the visual field at 25°/s. Luminance of the bright bar stimulus on black background was approximately 24 cd/m^2^. This stimulus was adjusted sequentially by either rotating the bar by π/8 radians or by halving the grey-value of the vertical bar to produce a stimulus set containing sixteen stimuli. All stimuli were given such that the centre of the 4° wide bar passed from the caudal to the rostral edge of the 25° visual field in one second. All image acquisition and stimulus presentation was controlled by μManager software^75^.

### Analysis of tectal responses

Small amounts of XY drift or motion artefacts from each tiff series were reduced by aligning each frame to its preceding frame using the ‘Rigid Body’ transformation in the StackReg plugin^76^ in ImageJ (United States National Institutes of Health). Using a custom-written MATLAB code, based on previous work by Panier and colleagues^77^, the tiff series was prepared for segmenting individual somata by first generating a mean intensity projection of the series. Detection of cell outlines was improved by applying a two-dimensional Laplacian of Gaussian filter followed by a morphological tophat transformation and Gaussian lowpass filter, each with a σ of approximately half an average cell diameter (7 pixels). The resulting images were then segmented into individual regions of interest (ROI) using the watershed function in MATLAB. This algorithm finds local minima in image intensity and the continuous peak in intensity surrounding this region is marked as its border. Only regions with an area of between 10 and 200 μm^2^, and with eccentricity of less than 0.9, were classified as cells.

In order to measure the activity of each neuron identified above, the baseline fluorescence of each ROI was determined by finding the 40^th^ percentile of its intensity over time (F0) prior to stimulus delivery. The raw intensity values at each time-point (Fi), minus this baseline, were then divided by the baseline fluorescence to yield a ΔF/F for each cell over time:


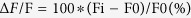


Significant neuronal firing was identified using a knowledge-based detection method, similar to that proposed by Patel and colleagues^78^. Specifically, the time-varying correlation coefficient between the fluorescence trace for each cell and an ‘example spike’ was calculated. The example spike was a 6 second trace created by averaging 50 user-defined calcium events. Calcium transients with a correlation coefficient greater than 0.7 to the example spike and a peak ΔF/F greater than 10% above baseline were classified as a neuronal response.

The proportion of active cells that were common to two given ensembles, relative to the total number of active cells across both assemblies, was determined using the matching index (MI) described by Romano and colleagues^44^. This was used to determine the repeatability of neuronal ensembles of the same functional cluster between trials, and to determine the presence of cells shared between neuronal ensembles belonging to different clusters in the same trial. The MI between two groups of cells was defined as twice the number of cells shared between both ensembles (X) divided by the total number of active cells in both ensembles (K):


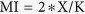


The orientation selectivity of cells was determined by averaging the peak response of the cell across all five trials to the stimulus at each orientation. For all orientations, the mean response (R_1_) was compared to that for the orthogonal orientation (R_2_) by the orientation selectivity index (OSI) using the formula:


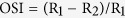


The maximum OSI, and the stimulus orientation to which it belongs, was then determined for each cell.

## Additional Information

**How to cite this article**: Thompson, A. W. and Scott, E. K. Characterisation of sensitivity and orientation tuning for visually responsive ensembles in the zebrafish tectum. *Sci. Rep*. **6**, 34887; doi: 10.1038/srep34887 (2016).

## Supplementary Material

Supplementary Information
