# Supplementary material for: Characterisation of sensitivity and orientation tuning for visually responsive ensembles in the zebrafish tectum
Source: Sci Rep. 2016 Oct 7;6:34887. doi: 10.1038/srep34887 (PMC5054398; doi:10.1038/srep34887)
Supplement: Supplementary Information [file srep34887-s1.pdf]

Characterisation of sensitivity and orientation tuning for visually responsive ensembles in the zebrafish tectum

Supplementary Information

Andrew W. Thompson[1]* and Ethan K. Scott[1, 2]*

[1]School of Biomedical Sciences, [2]The Queensland Brain Institute, The University of Queensland, St Lucia, QLD, 4072, Australia

Module	Component		Product	Manufacturer
Illumination Source	DPSS Laser		OBIS 488-150 LX	Coherent
Illumination Path	Laser launch		ST1XYS XY Translator	Thorlabs
			PY003 Pitch & Yaw Stage	
	Collimation and light fiber		F240FC-A NA=0.51	
			P1-460A-FC-1 Single Mode Fiber	
			F280FC-A NA=0.15	
	Beam Expander		LC1715 f=-50mm Concave Lens	
			LA1708 f=200mm Convex Lens	
	Beam Splitter		BS PLATE 50 X 50MM 50R/50T	Edmund Optics
	SPIM Path (x2)	Fixed Slit	6 x 50 MM Slit	Custom made
		Cylindrical Lens	LENS CYL 50 X 25MM X 75 FL VIS-NIR	Edmund Optics
		Illumination Objective	XLFLUOR4X 0.28NA	Olympus
	Illumination Plane Positioning	Vertical Translation Stage	70MM METRIC MIC Z-STAGE	Edmund Optics
		Horizontal Translation Stage	70MM SIDE METRIC MIC STAGE	
Detection Path	Detection Objective		XLUMPFLN 20XW 1.0NA, 2MM WD	Olympus
	Microscope Body	Filter	FF01-517/20-25	Semrock
		Tube Lens	Mounted AC508180A Ø2.0", efl=180mm	Thorlabs
	Camera		PCO-Edge 5.5	PCO
Specimen Positioning	X/Y-axis Translation Stage		XYT1/M	Thorlabs
	Z-axis Translation Stage		MTS50/M Z8	Thorlabs
Control Software	Image Acquisition & Stimulus Delivery		µManager	Vale Lab, UCSF

Supplementary Table 1. A list of components used in the construction of the light sheet microscope used in this study.

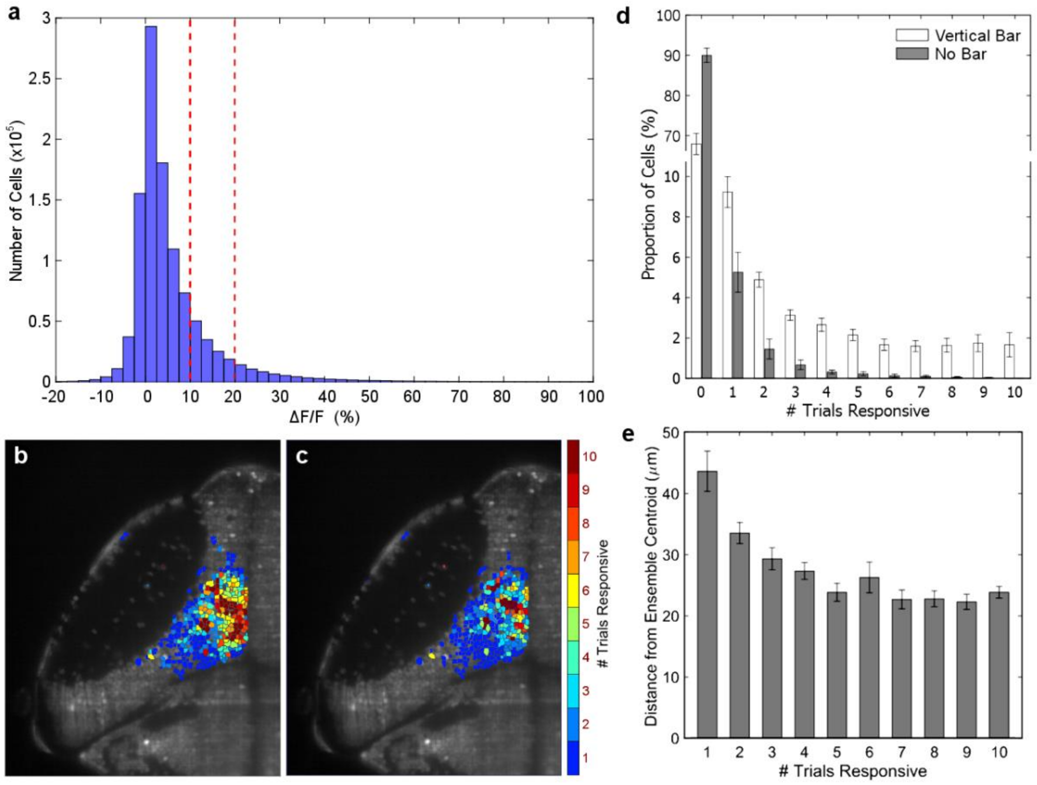

Supplementary Figure 1. Relative distribution of response frequencies within ensembles are unchanged using more stringent response thresholds.

(a) Histogram of response magnitudes for all cells across all trials responding to moving bar stimuli. The cutoff threshold for response used in this study was 10% ΔF/F (left dotted line). A more stringent threshold for response (20% ΔF/F, right dotted line) removed many small magnitude responses but did not alter the underlying descriptions of ensemble structure (see below). **(b)** The spatial distribution of cells with different response consistencies to the visual stimulus, from a single representative larva (as shown in Fig. 1f). **(c)** Based on a more stringent 20% ΔF/F response threshold, the spatial distribution of response consistencies was largely unchanged compared to the same cells in panel **b**, although fewer responses were registered because of the more stringent cutoff. **(d)** Using a 20% ΔF/F response threshold, the number of trials during which cells were active relative to no stimulus control shows the same divergence as in Fig. 1d, although the absolute number of cells was reduced. **(e)** With the more stringent 20% ΔF/F response threshold, cells with different response consistencies were located, on average, similar distances from the ensemble centroid as with the original threshold used in Fig. 1e.

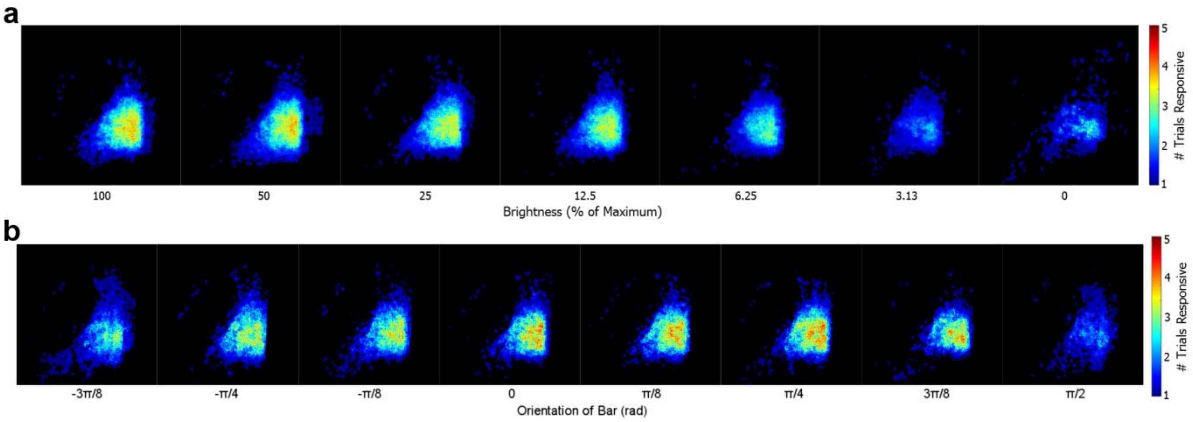

Supplementary Figure 2. Consistency of response frequencies within ensembles across multiple fish.

(a) Average distribution of visually-responsive neurons to presentations of moving vertical bar stimulus of decreasing brightness intensities from 18 experimental animals. Responsive cells within each fish were centered by their weighted centroid and overlaid across an average tectal template. **(b)** Average distribution of visually-responsive neurons to presentations of moving vertical bar stimulus of decreasing brightness intensities from 9 experimental animals. Responses were overlaid as for panel **a**.

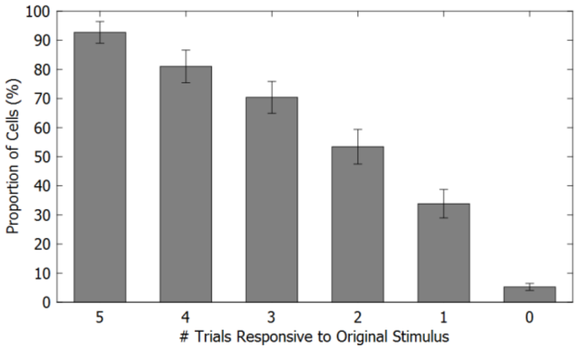

Supplementary Figure 3. Cells that were more frequently responsive to high strength stimuli remained more responsive to stimuli with lower intensity. Grey bars represent the proportion of cells that responded to the original, 100% brightness bar between zero and 5 times, that responded at least once to the 6.25% brightness bar. n = 18 larvae, error bars = SEM.

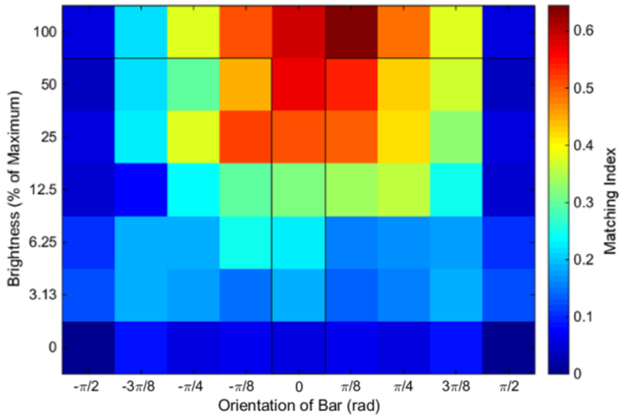

Supplementary Figure 4. The matching index comparisons between cells responding to all trials with the 14 different stimuli

Outlined in black, the Matching Indices report specifically on the proportion of cells from 100%, 0 rad condition that is also responsive to each other condition. As the absolute number of cells decreased with brightness, the corresponding matching index was also decreased. Similarly, as the orientation of the stimulus was rotated away from vertical, the matching index to the 100% brightness vertical bar was decreased, although it remained high until the π/2 stimulus, reflecting the cells' broad tuning. As the proportion of matching cells decreases in a similar manner across all columns, this suggests that cells from all orientations show similarly diminished responses to dimmer stimuli. These trends appeared consistent when comparing the composition of cells responsive during all different conditions.
